# Supplementary material for: Tead1a Initiates Transcriptional Priming Through the TEAD1a/YAP‐Notch1‐Spi1/Cebpα Axis to Promote Neutrophil Fate
Source: Adv Sci (Weinh). 2025 Aug 28;12(41):e05441. doi: 10.1002/advs.202505441 (PMC12591127; doi:10.1002/advs.202505441)
Supplement: Supplementary file 4 — Supplementary Dataset [file ADVS-12-e05441-s004.zip › 3-6.zip!/3-6/02-6.cluster/cluster16/Enrich/ego_MF.pdf]

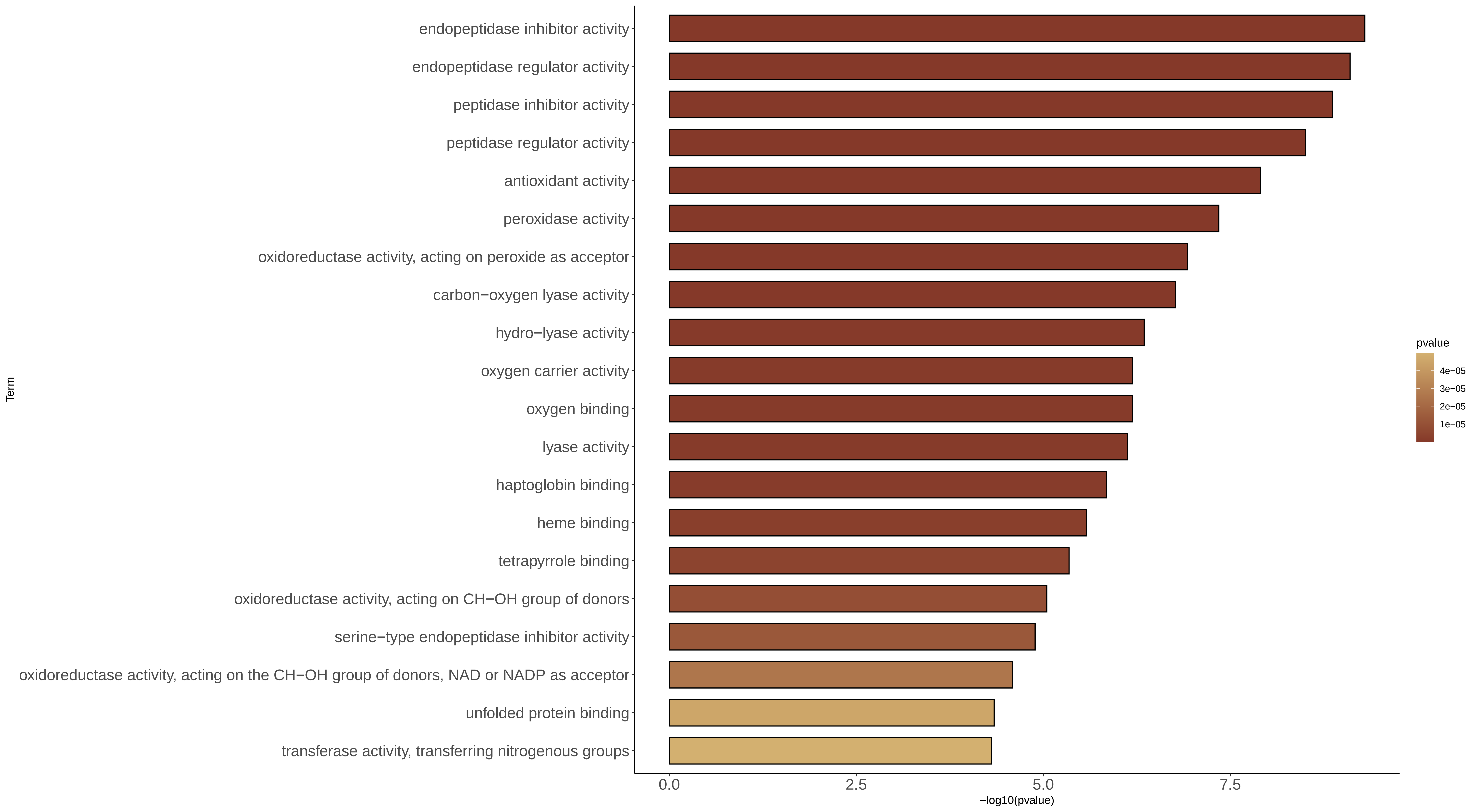

endopeptidase inhibitor activity
endopeptidase regulator activity
peptidase inhibitor activity
peptidase regulator activity
antioxidant activity
peroxidase activity
oxidoreductase activity, acting on peroxide as acceptor
carbon−oxygen lyase activity
hydro−lyase activity
oxygen carrier activity
oxygen binding
lyase activity
haptoglobin binding
heme binding
tetrapyrrole binding
oxidoreductase activity, acting on CH−OH group of donors
serine−type endopeptidase inhibitor activity
oxidoreductase activity, acting on the CH−OH group of donors, NAD or NADP as acceptor
unfolded protein binding
transferase activity, transferring nitrogenous groups
Term
0.0
2.5
5.0
7.5
−log10(pvalue)
pvalue
4e−05
3e−05
2e−05
1e−05
